# Co-Cultures of *Pseudomonas aeruginosa* and *Roseobacter denitrificans* Reveal Shifts in Gene Expression Levels Compared to Solo Cultures

**DOI:** 10.1100/2012/120108

**Published:** 2012-04-01

**Authors:** Crystal A. Conway, Nwadiuto Esiobu, Jose V. Lopez

**Affiliations:** ^1^Oceanographic Center, Nova Southeastern University, Dania Beach, FL 33004, USA; ^2^Department of Biological Sciences, Florida Atlantic University, Davie, FL 33314, USA

## Abstract

Consistent biosynthesis of desired secondary metabolites (SMs) from pure microbial cultures is often unreliable. In a proof-of-principle study to induce SM gene expression and production, we describe mixed “co-culturing” conditions and monitoring of messages via quantitative real-time PCR (qPCR). Gene expression of model bacterial strains (*Pseudomonas aeruginosa* PAO1 and *Roseobacter denitrificans* Och114) was analyzed in pure solo and mixed cocultures to infer the effects of interspecies interactions on gene expression *in vitro*, Two *P. aeruginosa* genes (*PhzH* coding for portions of the phenazine antibiotic pathway leading to pyocyanin (PCN) and the *RhdA* gene for thiosulfate: cyanide sulfurtransferase (Rhodanese)) and two *R. denitrificans* genes (*BetaLact* for metallo-beta-lactamase and the *DMSP* gene for dimethylpropiothetin dethiomethylase) were assessed for differential expression. Results showed that *R. denitrificans DMSP* and *BetaLact* gene expression became elevated in a mixed culture. In contrast, *P. aeruginosa* co-cultures with *R. denitrificans* or a third species did not increase target gene expression above control levels. This paper provides insight for better control of target SM gene expression *in vitro* and bypass complex genetic engineering manipulations.

## 1. Introduction

Interactions among diverse microbial species are dynamic and most likely propel many of the adaptations that allow the occupation of diverse niches that can range from biofilms to host digestive tracts to multiple marine habitats [1, 2]. These interactions among diverse bacteria can be either beneficial such as in symbioses with eukaryotic hosts [3, 4] or antagonistic due to competition within multiple species microcosms [5].

Although necessary for identification and certain microbiological experiments, traditional bacteriological methods which focus on isolating microbes as axenic cultures do not provide much insight into the ecological dynamics of natural habitats, where microbes thrive and interact with different species and within complex communities. Interspecies interactions involve the action of multiple genetic and metabolic pathways which can result in mutualistic or antagonistic bacterial effects. The molecular basis of some ecological interactions have been linked to secondary metabolites (SMs, also known as “natural products”), which are organic, biosynthesized compounds such as antibiotics and toxins not essential for basic growth or reproduction in organisms. SMs are used for defense, chemical signaling, and host-microbe interactions [6, 7]. SMs may stem from overflow products or evolutionary relics of former physiological functions [2]. Many unique and biologically active SMs continue to increase the interest of academic and industrial researchers [8]. However, pure cultures of microbes often fail to yield reliable or consistent biosynthesis of SMs [9].

Current studies on SM gene expression often rely on expensive, recombinant cloning of target genes into heterologous microbes or large-scale genomic sequencing projects [10]. An alternative experimental strategy would be to induce, measure, and track the expression of microbial SM genes while they grow in mixed culture conditions to better mimic antagonism and interaction in a natural environment. Applying the latter approach to well-studied model bacteria may lead to the elucidation of gene expression patterns from lesser known, nonmodel microbial organisms lacking genomic sequence data.

Based on a primary tenet of bacterial antagonism [5], we now report that targeted SM genes from model bacteria can be reproducibly induced after challenging these microbes *in vitro*. Here, the variable stressor is the “co-culturing” process of marine microbes (defined as growth of >1 bacterial species within one flask). Secondly, levels of specific gene expression were tracked and quantified by quantitative real-time PCR (qPCR) [11]. Model bacteria, *Pseudomonas aeruginosa* and marine *Roseobacter denitrificans*, were chosen for this study due to their available complete genome sequences and possible roles in defense and secondary metabolism [12–14]. Moreover, *Pseudomonas aeruginosa* is a human pathogen, and the particular PAO1 strain has been found in the marine environment playing an important role in biofilms.

 This proof-of-principle study now presents specific and reproducible results showing that the selected bacterial genes can be induced by the act of co-culture mixing. Although we did not directly measure each corresponding gene product with chemical methods, the detection of expressed mRNA transcripts serves as a proxy for potential SM production. Moreover, the induced gene expression patterns clearly differ from solo pure cultures.

## 2. Materials and Methods

The microbial taxa, *Pseudomonas aeruginosa* PAO1 and *Roseobacter denitrificans *Och114, were chosen because they are well-characterized microbial species and can occur in marine habitats, which is a focus of our laboratory. These strains were provided by the Arizona State University (ASU) and the PathoGenesis Corporation, respectively.

All cultures and co-cultures were grown in marine broth before and after mixing and sampled for standard RNA extraction at the different time points indicated. RNA was isolated using the RNeasy Mini Kit (Qiagen, Valencia, CA) following the manufacturer's protocol. Dual co-cultures of *P. aeruginosa-R. denitrificans* were tracked by quantitative real-time PCR (qPCR) utilizing SYBR green detection [15].

Four genes from the two model bacterial genomes, *Pseudomonas aeruginosa* (GenBank AE004091) and marine *Roseobacter denitrificans* (GenBank CP000362) were retrieved and used for gene-specific primer design: *PhzH, RhdA, BetaLact, *and* DMSP* (Table 1). The qPCR primers were designed using PRIMER BLAST from the National Center for Biotechnology Information website (http://www.ncbi.nlm.nih.gov/tools/primer-blast/).

Expressions of the same genes in solo and co-cultures of *P. aeruginosa* and *R. denitrificans* were compared (first column set to 1.0). In this study, the solo culture with the target genes acted as the control. Then, the Ct values of both the control and the genes in question were normalized to the *P. aeruginosa* housekeeping gene (*RNA polymerase*). All qPCR runs were performed as triplicate reactions with the same DNA template and gene-specific primers, on a single 48-well plate which also included negative (zero DNA) controls.

After qPCR amplification the comparative threshold method (ΔΔCt analysis) was applied to evaluate the relative changes in gene expression from qPCR experiments [16]. Computer programs GeneX (Bio-Rad) and Excel (Microsoft) were used to calculate the equation: [delta][delta]Ct = [delta]Ct_, sample_–[delta]Ct_,  reference_(BioRad).

## 3. Results and Discussion

### 3.1. Gene Expression Analyses of Solo and Co-Cultures with qPCR

Both *P. aeruginosa* and *R. denitrificans *entered log phase at six hours and were then combined for co-culturing and subsequent gene expression analyses throughout log phase. A housekeeping gene,* DNA directed RNA polymerase (RNA pol), subunit alpha* expression appeared constant throughout all qPCR runs meaning their expression level was unaffected by the experimental conditions (data not shown). A third species, *Salinispora arenicola (*provided by the Joint Genome Institute), was originally intended for SM gene tracking but because of disparate growth patterns was only used for co-culture antagonism.

**Figure 1:**
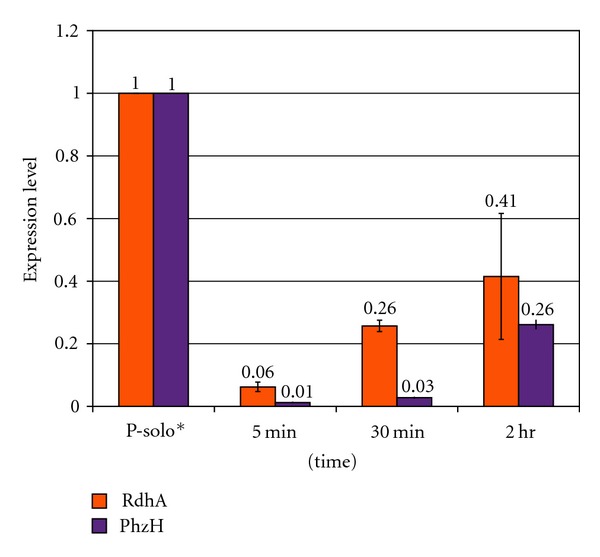
*P. aeruginosa *gene expression in the *P. aeruginosa-R. denitrificans R. denitrificans *co-cultures. Relative gene expression levels of *P. aeruginosa RdhA *and* PhzH *genes in dual co-cultures of *P. aeruginosa-R. denitrificans *were determined by quantitative real-time PCR (qPCR) with the SYBR green method [15].

Figure 1 indicates that the act of co-culturing *P. aeruginosa*-*R. denitrificans* strains caused a measureable effect, as both *P. aeruginosa RdhA* and *PhzH *genes showed overall lower gene expression relative to control solo culture levels (Figure 1). A similar effect of lower *RdhA* gene expression was observed in triplet (*P. aeruginosa-R. denitrificans-S. arenicola*) co-cultures (data not shown). 

**Figure 2:**
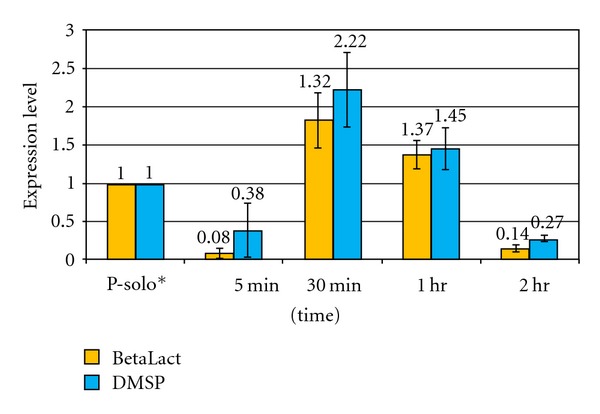
*R. denitrificans BetaLact *and *DMSP *gene expression in *R. denitrificans-P. aeruginosa *dual co-culture. Methods were as described in Figure 1 except for the fourth time point added at one hour.

 By contrast, *R. denitrificans BetaLact *and *DMSP *genes showed different patterns including repressed and escalated levels of gene expression across different time points and co-cultures. For example, in *R. denitrificans-P. aeruginosa* dual co-cultures, (Figure 2) both *BetaLact *and *DMSP *gene expression appeared lower than solo levels at initial mixing but then rose by about 2.0 fold after 30 minutes, and then leveled off. At two hours the gene expression levels of both genes decreased below solo culture level.

**Figure 3:**
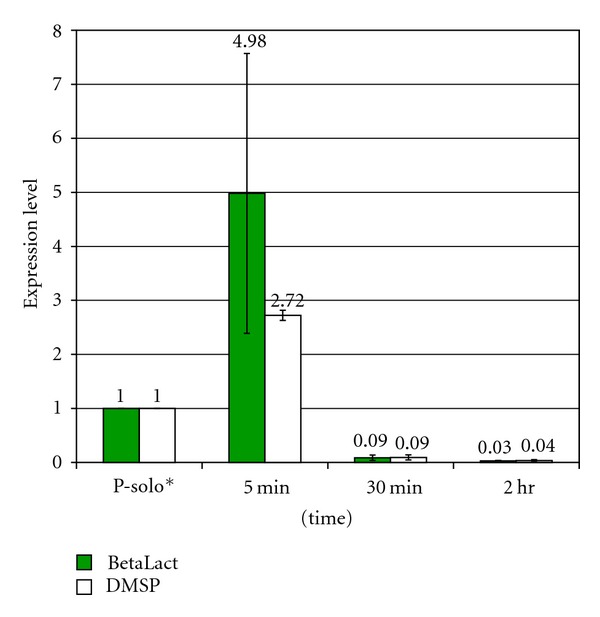
*R. denitrificans BetaLact *and *DMSP *relative gene expression in *R. denitrificans-S. arenicola *dual co-culture. Methods were as described in Figure 1.

In a *R. denitrificans-S. arenicola* dual co-culture (Figure 3), the same *R. denitrificans BetaLact *and *DMSP* genes exhibited a large 2.7–4.0-fold increase of gene expression with a more rapid onset after initial mixing of the two bacteria. The *DMSP* gene is expressed about twice as much as the *R. denitrificans* solo culture, but lower than *BetaLact* gene expression which is expressed about three times as much as the *R. denitrificans* solo culture. Similar increases were observed in duplicate experiments. A decrease in the expression of both genes occurred after more than 30 minutes of co-culturing.

## 4. Discussion

### 4.1. Gene Expression Patterns Seen in P. aeruginosa and R. denitrificans Co-Cultures

The primary aim of this research was achieved by showing that gene expression of certain targeted genes could be reproducibly induced or affected by systematic co-culturing in multistrain growth conditions. As mentioned above, the choice of genes generally centered on “secondary metabolism” (SM). Because some SMs show therapeutic potential or bioactive effects, large-scale efforts involving more sophisticated biotechnologies have been initiated in recent years to characterize and exploit the rich biochemical and genetic diversity within secondary metabolite producing organisms [17]. For example, with the advances in recombinant DNA technology, efforts have focused on the cloning and sequencing of complete polyketide biosynthetic gene loci (which can be very large) with the expectation of expressing these metabolic pathways in a foreign, heterologous host [6, 7]. The recent creation of a synthetic microbial cell [18] also conforms to an ultimate goal of controlling gene expression through artificial constructs.

Interestingly, with respect to our focus on gene expression,* P. aeruginosa* contains the highest proportion of regulatory genes observed in a microbial genome [13]. Some *P. aeruginosa *strains have diverse antimicrobial activities in different types of marine invertebrates, such as sponges [19]. In *P. aeruginosa, *we focused on the *PhzH *gene, which codes for the production of phenazine-1-carboxamide derived from the common precursor, phenazine-1-carboxylic acid (PCA) [20]. Phenazines are biologically versatile compounds involved in microbial competition, suppressing soil plant pathogens, and virulence in human and animal hosts [21]. A second targeted gene is *RhdA*, a thiosulfate: cyanide sulfurtransferase (Rhodanese) [22]. The *RhdA* gene products in *P. aeruginosa* protect the microbe from cyanide toxicity by converting the cyanide to the less toxic form of thiocyanate.

In co-culture experiments, the *P. aeruginosa RdhA *gene exhibited generally higher and more variable and inducible expression levels compared to *P. aeruginosa PhzH *gene. In contrast, *PhzH *gene expression appeared suppressed throughout most sample time points and never had its gene expression levels higher than its solo culture. However, the gradual increase is consistent with previous studies showing that PCN products appear mostly in late log phase [21].

*Roseobacter denitrificans *Och114 is a purple marine aerobic anoxygenic phototroph (AAP) [14] that plays an inimitable role in global energy and carbon cycles. A unique trait of this bacteria is that they are able to grow both photoheterotrophically (in the presence of oxygen) and anaerobically (in the dark using nitrate as an electron acceptor) [14]. *R. denitrificans* belongs in a bacterial clade with diverse metabolism, including its designation as one of the first bacteria characterized exhibiting anoxygenic phototrophic features [23].

*R. denitrificans *genes exhibited much different expression patterns compared to *P. aeruginosa*, with very large gene response spikes to co-culture conditions. Both *R. denitrificans *genes were expressed at higher levels than controls and interestingly behaved in a parallel fashion that tracked each other's rise and fall of expression levels throughout all time periods. *R. denitrificans* metallo-beta-lactamases provide resistance against beta-lactam antibiotics, which account for more than half of the world's antibiotic market. DMSP lyase catalyzes the creation of DMS (dimethyl sulfide) and acrylate from DMSP (dimethylsulfoniopropionate). No exact function of DMSP has been discovered to date, but it has been hypothesized that DMSP provides osmoregulation, some protection from oxidative stress, and herbivory. *R. denitrificans* showed the widest changes in SM gene expression levels throughout the study, possibly because the secondary metabolite genes chosen for this organism are not needed in high levels when in these particular co-cultures. *R. denitrificans BetaLact* gene expression levels rose even though *P. aeruginosa* is not known to produce any beta-lactam antibiotics. This opens the possibility that other SM genes of *R. denitrificans *could be activated upon co-culturing, even though not directly related to defense or antagonism *per se*.

When in a community, microbes will compete with other microbes for both resources and space [24]; for example both *P. aeruginosa* and *R. denitrificans* are competitive microbes, both with strong abilities to outcompete and kill other microorganisms [13, 14, 21]. The spike in gene expression of* R. denitrificans *co-cultures may stem from a defensive reaction to the presence of the second microorganism (Figures 2 and 3). Alternatively since both bacterial species can potentially coexist in diverse environments, lower levels of *P. aeruginosa* SM gene expression observed in the *P. aeruginosa-R. denitrificans* co-culture may be due to relative acclimatization to each other.

### 4.2. Possible Quorum Sensing in Co-Culture

Although not measured directly *per se*, quorum sensing (QS) factors may have played roles in co-culture gene expression in this study [25]. Bacterial QS compounds change the physiology of conspecific members of the population and represent one other possible explanation for the changes in gene expression during co-culture [26–28]. Throughout the past decade it has become increasingly recognized that bacteria are capable of intercellular communication moderated by QS factors such as autoinducers, or derivatives of homoserine lactone which facilitate adaptations to changing environmental conditions based on the population density of the producing microorganism [29]. This phenomenon probably includes regulating the expression or repression of secondary metabolites [25], which can affect degrees of cooperation or antagonism within and between different species, respectively [5, 26].

In this context, the abrupt decrease in *R. denitrificans *gene expression observed in the *P. aeruginosa*-*R. denitrificans *dual co-culture (Figure 2) may have stemmed from an interruption in *R. denitrifican* quorum sensing abilities after the initial mixing of the solo bacterial populations. That is, any quorum sensing factors released by the single species became diluted by at least half upon co-culturing. Once the concentration factors fell below minimum threshold levels, they may have lost their ability to affect or maintain the levels of intraspecies signaling present in each solo culture before the mixing. This could represent a switch between intra-specific cooperation and interspecies antagonism.

Secondly, it is quite possible that bacterial interactions in mixed cultures (i.e., in nature) involve the degradation or modification of QS factors secreted by other members of the community. This would result in repression of some gene products, as they are degraded by one of the bacteria. Phenazine PCN participates in a complex pathway of QS regulation [20], and therefore we acknowledge that sufficient explanation for *PhzH* gene expression levels requires further experimentation. Alternatively, none of the target genes may be under QS control.

Although other possible explanations remain, this study shows that varying, yet reproducible, expression levels appear to be gene specific and context dependent. Also, specific gene induction appeared temporary but clearly resulted from the act of mixed species co-culturing. This paper points to future studies and experimental strategies that can focus on factors affecting the structure of artificial or more complex microbial communities and interactions [4]. Finally, finding specific molecules or signals that control unique secondary metabolite pathways and their genes may have wider ramifications for natural products research, microbial ecology and the pharmaceutical industry.

## Figures and Tables

**Table 1:** Primer sequences used in Quantitative PCR analyses of gene expression of target bacteria in solo and co-cultures.

Target gene	Abbreviation	Source species	Primer sequences, 5′ to 3′	Gene product length
DNA directed RNA polymerase, subunit alpha	*HGK*	*P. aeruginosa*	TGATTTCGGTCAGGGACTTC GATGACCTGGAACTGACCGT	139
DNA directed RNA polymerase, subunit alpha	*HGK*	*R. denitrificans*	TCACCTCTGTGCAGATCGAC TGTCACCAGCAGTCACAACA	177
Thiosulfate:cyanide sulfurtransferase (Rhodanese)	*RdhA*	*P. aeruginosa*	AGGAAGTGATCACCCACTGC CTCTACAGGGGTATCGGGGT	140
Biosynthesis of pyocyanin	*PhzH*	*P. aeruginosa*	TGCGCGAGTTCAGCCACCTG TCCGGGACATAGTCGGCGCA	214
Metallo-beta-lactamase	*BetaLact*	*R. denitrificans*	AATACGAATTGCCCAGCATC GCAGGCCATAACAACAACCT	184
Dimethylpropiothetin dethiomethylase	*DMSP*	*R. denitrificans*	GTGCCGCACTGGCTGTGGAT	125
